# Genotype-Specific Expression of Selected Candidate Genes Conferring Resistance to Leaf Rust of Rye (*Secale cereale* L.)

**DOI:** 10.3390/genes15030275

**Published:** 2024-02-22

**Authors:** Rumana Azad, Tomasz Krępski, Mateusz Olechowski, Bartosz Biernacik, Magdalena Święcicka, Mateusz Matuszkiewicz, Marta Dmochowska-Boguta, Monika Rakoczy-Trojanowska

**Affiliations:** 1 Department of Plant Genetics, Breeding and Biotechnology, Institute of Biology, Warsaw University of Life Sciences, Nowoursynowska 166, 02-787 Warszawa, Poland; rumanaazadbd@gmail.com (R.A.); tomasz_krepski@sggw.edu.pl (T.K.); mateuszolechowski1@gmail.com (M.O.); bartosz_biernacik@sggw.edu.pl (B.B.); magdalena_swiecicka@sggw.edu.pl (M.Ś.); mateusz_matuszkiewicz@sggw.edu.pl (M.M.); 2 Plant Breeding and Acclimatization Institute—National Research Institute, Radzikow, 05-870 Blonie, Poland; m.dmochowska-boguta@ihar.edu.pl

**Keywords:** *Puccinia recondita* f. sp. *secalis*, winter rye, seedling stage resistance, resistance genes, quantitative real-time PCR

## Abstract

Leaf rust (LR) caused by *Puccinia recondita* f. sp. *secalis* (*Prs*) is a highly destructive disease in rye. However, the genetic mechanisms underlying the rye immune response to this disease remain relatively uncharacterised. In this study, we analysed the expression of four genes in 12 rye inbred lines inoculated with *Prs* at 20 and 36 h post-treatment (hpt): *DXS* (1-deoxy-D-xylulose 5-phosphate synthase), *Glu* (β-1,3-glucanase), *GT* (UDP-glycosyltransferase) and *PR-1* (pathogenesis-related protein 1). The RT-qPCR analysis revealed the upregulated expression of the four genes in response to *Prs* in all inbred lines and at both time-points. The gene expression data were supported by microscopic and macroscopic examinations, which revealed that eight lines were susceptible to LR and four lines were highly resistant to LR. A relationship between the infection profiles and the expression of the analysed genes was observed: in the resistant lines, the expression level fold changes were usually higher at 20 hpt than at 36 hpt, while the opposite trend was observed in the susceptible lines. The study results indicate that *DXS*, *Glu*, *GT* and *PR-1* may encode proteins crucial for the rye defence response to the LR pathogen.

## 1. Introduction

Rye (*Secale cereale* L.), which is an economically important crop mainly grown in Central and Eastern Europe, is highly tolerant to abiotic and biotic stresses, but it is still affected by 36 fungal and 2 bacterial diseases (https://www.apsnet.org/edcenter/resources/commonnames/Pages/Rye.aspx; accessed on 15 December 2023). Among these fungal diseases, rusts, including leaf rust (LR), stem rust and yellow rust caused by the obligate, biotrophic basidiomycetous fungi *Puccinia recondita* f. sp. *secalis* (*Prs*), *Puccinia graminis* f. sp. *secalis* and *Puccinia striiformis* var. *striiformis*, respectively, can reduce rye yields by up to 70% [1]. Yield losses due to LR alone can reach 40% under natural conditions [2]. To date, 16 dominant *Pr* genes (*Pr1-5*, *Pr-d–f*, *Pr-i–l*, *Pr-n*, *Pr-p*, *Pr-r* and *Pr-t*) distributed on five of the seven rye chromosomes (1R, 2R, 4R, 6R and 7R) in blocks of linked genes responsible for the resistance to *Prs* clones have been identified using Mendelian methods [3,4,5]. Most of the *Pr* genes (*Pr1-5*, *Pr-d–f*, *Pr-n* and *Pr-r*) confer resistance to a broad range of single-pustule isolates, with *Pr1*, *Pr2*, *Pr-d* and *Pr-r* reportedly being very useful for rye breeding [3]. The recent publication of two rye genome sequences (Lo7 and Weining) [6,7] enabled Vendelbo et al. [8,9] to precisely characterise LR-related genes at the molecular level. Specifically, five LR resistance-associated quantitative trait loci (QTLs) were mapped on chromosome arms 1RS, 1RL, 2RL, 5RL and 7RS on the basis of a genome-wide association study. Two QTLs located on chromosome arms 1RS and 7RS were revealed to be crucial for LR resistance. The most important resistance-associated marker on chromosome arm 1RS is physically co-localised with molecular markers delimiting the previously characterised *Pr3* gene. The second region on 7RS comprises many nucleotide-binding leucine-rich repeat (NLR) genes, one of which, provisionally named *Pr6*, is similar (in terms of the encoded protein) to the wheat gene (*Lr1*) conferring LR resistance located on chromosome arm 5DL. Unfortunately, these earlier studies did not investigate the expression of the identified genes. A previous study by Święcicka et al. [10] examined the expression of rye genes affected by LR and determined these genes control the benzoxazinoid biosynthesis pathway in three inbred lines (D33, D39 and L318; the last one was also included in this study). In this earlier study, the expression of one of the examined genes, *ScBx4*, which encodes a cytochrome P450 monooxygenase, was induced in infected plants at all time-points (8, 17, 24 and 48 h post-treatment). The single-nucleotide polymorphism in this gene (ScBx4_1583) is stably associated with the field resistance of adult plants to LR, which is consistent with the findings of another study [11]. Accordingly, this gene contributes to non-race-specific resistance in adult plants and seedlings.

By conducting an RNA-seq analysis, we recently identified hundreds of differentially expressed genes following a *Prs* infection [12]. We selected four of these genes, namely *SECCE1Rv1G0055210*, *SECCE6Rv1G0429310*, *SECCE7Rv1G0520220* and *SECCE7Rv1G0464120*, which respectively encode 1-deoxy-D-xylulose 5-phosphate synthase (DXS), β-1,3-glucanase (Glu), UDP-glycosyltransferase (GT) and pathogenesis-related protein 1 (PR-1); these genes are characterised by high BaseMean values and fold-change (FC) values. The expression levels of all of the selected genes are upregulated by a *Prs* infection.

Although *DXS* affects the resistance to various diseases, such as late blight in potatoes [13] or downy mildew in spinach [14], whether it mediates resistance to rusts is unclear. In contrast, the involvement of the following three genes in plant immune responses to rusts, including LR, has been confirmed by many studies: *Glu* [15,16,17], *GT* [10,18,19,20] and *PR1* [15,17,21,22,23]. However, whether *DXS*, *Glu*, *GT* and *PR-1* protect rye against LR (or other fungal diseases) remains unknown. Therefore, in this study, we examined the expression of these genes by performing a quantitative real-time PCR (RT-qPCR) analysis to assess their potential role in the rye immune response to LR.

## 2. Materials and Methods

### 2.1. Plant Materials

The plant materials used in this study consisted of 12 rye inbred lines (Table 1).

### 2.2. Experimental Design

Seeds from the 12 rye inbred lines were germinated on tissue/filter paper moistened with distilled water in Petri dishes and incubated at 22 °C for 2 days in the dark. All seedlings were transferred to 12 cm diameter plastic pots (10 seedlings per pot) filled with sterilised peat substrate and then incubated in a growth chamber at 22 °C with a 16 h light (illumination intensity of 60 µmol m^−2^ s^−1^)/8 h dark photoperiod, 50% humidity, for 10 days. Experiments were conducted with three biological replicates, each comprising one pot with 10 plants and two time-points [20 and 36 h post-treatment (hpt)] that were the same as in our previous work [24].

### 2.3. Inoculation of Rye Seedlings with Prs and the Mock Control

To inoculate 12-day-old rye seedlings, *Prs* isolate 1.1.6 derived from a single urediniospore (selected based on a preliminary experiment involving detached-leaf inoculations with 30 isolates; [10]) was re-suspended in Novec-7100 (3M, St. Paul, MN, USA) engineered fluid (at a density of 1 mg cm^−3^). Plants were inoculated using brown glass diffusers (Roth, Basel, Switzerland). The same method and equipment were used for the mock inoculation of the control plants with Novec-7100 engineered fluid alone. The inoculated plants were incubated for 24 h in black boxes at 18 °C with 100% humidity, after which they were incubated under the same conditions as before the inoculation as previously described by Dmochowska-Boguta et al. [25].

### 2.4. Microscopic Analysis

Three days after plants were inoculated with *Prs*, the infected leaves were harvested, transferred to a fixative solution consisting of ethanol–dichloromethane (POCH, Gliwice, Poland), (3:1) and 0.15% trichloroacetic acid (ROTH, Karlsruhe, Germany) and incubated for 24 h. The whole leaf samples were stained with calcofluor white (Fluorescent brightener 28; Sigma F-6259, Milwaukee, WI, USA) as described by Orczyk et al. [26] and then transferred to a glycerol solution containing lactophenol [phenol (POCH, Gliwice, Poland): glycerol (ROTH, Karlsruhe, Germany): lactic acid (POCH, Gliwice, Poland); 1:1:1 v/v/v]. A Diaphot fluorescence microscope (Nikon Diaphot, Aizu, Japan, epifluorescence optics with excitation 340–380 nm, barrier filter 420 nm and dichroic mirror 400 m) was used to examine the stained whole leaves for the presence of the following infection profiles: (i) appressorium + haustorium mother cell (HMC); (ii) appressorium + HMC + micronecrosis; and (iii) appressorium + micronecrosis.

### 2.5. Macroscopic Analysis

Ten days after the *Prs* inoculation, a macroscopic examination was completed to evaluate the infection types using the following 0–4 Murphy Scale [27]: 0 = immune (no visible reaction); 1 = resistant (minute uredinia surrounded by chlorosis or necrosis); 2 = moderately resistant (small pustules surrounded by chlorosis); 3 = moderately susceptible (moderately large pustules surrounded by chlorosis); and 4 = susceptible (moderately large to large pustules with little or no chlorosis).

### 2.6. Total RNA Extraction and cDNA Synthesis

Total RNA was isolated from 100 mg of the aerial parts of the *Prs*- and mock-inoculated (control) rye seedlings using the GeneMATRIX Universal RNA Purification Kit (Eurx, Gdańsk, Poland). The isolated RNA was eluted using 50 μL RNase-free water and stored at −80 °C. The quality of the extracted RNA was assessed using an ND-2000 spectrophotometer (NanoDrop; Thermo Fisher Scientific, Waltham, MA, USA) to calculate two wavelength ratios (A260/230 and A260/280 nm). The integrity of the RNA was checked by 1.5% agarose gel electrophoresis. The RNA was treated with Turbo DNase (Thermo Fisher Scientific, Waltham, MA, USA) to eliminate any DNA contaminants. The first cDNA strand was synthesised from 1 μg total RNA using the RevertAid First Strand cDNA Synthesis Kit (Thermo Fisher Scientific, Waltham, MA, USA).

### 2.7. Quantitative Real-Time PCR Analysis

The *DXS*, *Glu*, *GT* and *PR-1* genes respectively encoding 1-deoxy-D-xylulose 5-phosphate synthase, β-1,3-glucanase, UDP-glycosyltransferase and pathogenesis-related protein 1 were selected on the basis of the results of RNA-seq analyses [12] employing three rye inbred lines: D33, D39 and L318. The two first lines were infected with the same *Prs* isolate (1.1.6) as in this work, so they are not included here. However, the third line was infected with a different isolate, and therefore, it was used in the current analysis. The sequencing data were deposited in the SRA database (http://www.ncbi.nlm.nih.gov/bioproject/888031 (accessed on 25 January 2024); BioProject accession number: PRJNA888031). These four genes were selected because of their high BaseMean and FC values as well as their consistent upregulated expression levels in most cases. The details of RNA-seq analysis are described by Krępski et al. [24].

The relative expression levels of these genes were determined by an RT-qPCR analysis, which was completed using a 96-well plate, with three biological replicates and two technical replicates. Each experimental setup contained two of the four target genes and two reference genes, namely *Act* and *ADP* respectively encoding actin and ADP-ribosylation factor 1-like protein (Table 2). The RT-qPCR analysis was performed using a LightCycler 96 Real Time System (Roche, Basel, Switzerland). Each 20 μL reaction volume consisted of 8 μL cDNA (20 ng), 1 μL each gene-specific primer (10 mM) (Table 2) and 10 μL FastStart Essential DNA Green Master (Roche). The PCR program was as follows: 95 °C for 600 s; 35 cycles of 95 °C for 10 s, 57 °C for 10 s and 72 °C for 15 s; 95 °C for 10 s, 55 °C for 60 s and melting curve analysis (95 °C for 10 s, 65 °C for 60 s and 97 °C for 1 s). Gene expression levels were normalised against the expression levels of the two reference genes *Act* and *ADP* according to the 2^−ΔΔCt^ method [28]. The statistical analysis of differentially expressed genes was performed using the REST software [29], with *p* ≤ 0.05 set as the threshold for significance. Graphs were prepared using the OriginPro^®^ 2023 software.

## 3. Results

### 3.1. Microscopic and Macroscopic Analyses of LR Symptom Development

**Figure 1:**
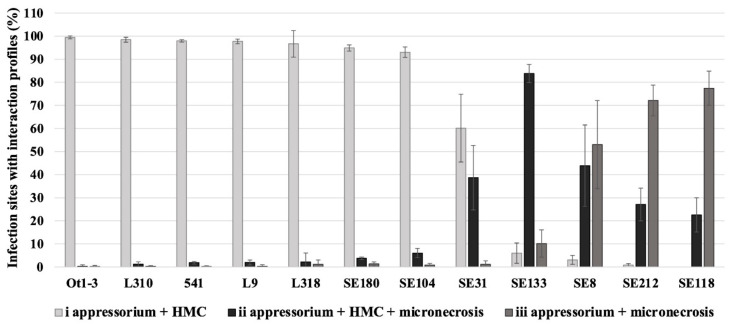
Microscopic examination of rye seedlings—*Prs* 1.1.6 isolate interactions at 3 days post-inoculation. The results show the average percentage of infection sites with profiles i–iii, with standard deviation. Observations were made for an average of 105 infection sites per leaf sample in three–four replicates. Bar colours: light grey—profile i (appressorium + HMC); black—profile ii (appressorium + HMC + micronecrosis); dark grey—profile iii (appressorium + micronecrosis).

Microscopic evaluation at 3 days post-inoculation allows us to see the most differences between susceptible and resistant lines, which would not yet be visible at 20 and 36 h post-inoculation and would no longer be visible at later days post-inoculation. Three infection profiles were used to describe the rye–LR interaction. In lines Ot1-3, L310, 541, L9, L318, SE180 and SE104, the infection sites mainly contained HMCs; profile i was detected at 93.07–99.47% of the infection sites. In contrast, necrosis was observed at more infection sites in line SE31; profile ii was detected at 38.69% of the infection sites of SE31. In the remaining lines, necrosis was detected at most of the infection sites (profiles ii and iii). In line SE133, profiles ii and iii were detected at 83.84% and 10.17% of the infection sites, respectively. In lines, SE8, SE212 and SE118, profile iii (i.e., necrosis without HMCs) was the most common profile at the infection sites (53.01%, 72.12% and 77.44%, respectively). The results of the microscopic examination are presented in Figure 1.

**Figure 2:**
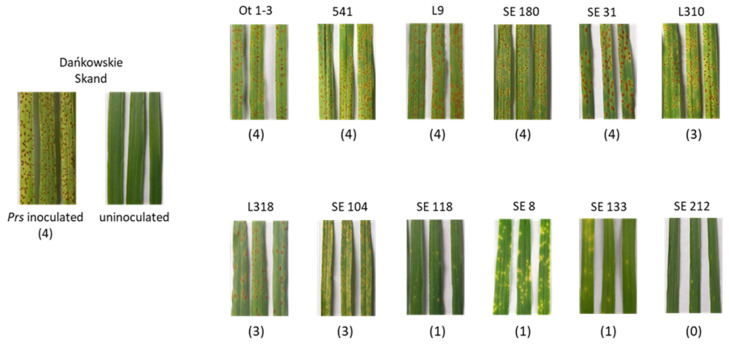
Macroscopic examination of LR symptoms 10 days after the inoculation with isolate 1.1.6. Macroscopic evaluation of LR symptoms, 10 days after inoculation with 1.1.6 isolate. In brackets—infection types based on the 0–4 Murphy Scale [27]: 0 = immune (no visible reaction); 1 = resistant (minute uredinia surrounded by chlorosis or necrosis); 3 = moderately susceptible (moderately large pustules surrounded by chlorosis); and 4 = susceptible (moderately large to large pustules with little or no chlorosis). The infection type 2 = moderately resistant (small pustules surrounded by chlorosis) was not observed. Dańkowskie Skand—susceptible control inoculated with *Prs* and uninoculated.

The results of the microscopic examination were supported by the macroscopic analysis (Figure 2; Table S2). This analysis was performed 10 days post-inoculation when macroscopic differences were already visible. Specifically, the infection profiles determined using the microscope were in accordance with the observed symptoms 10 days after plants were inoculated with *Prs*; the only exception was line SE31.

### 3.2. Analysis of DXS, Glu, GT and PR-1 Expression

**Figure 3:**
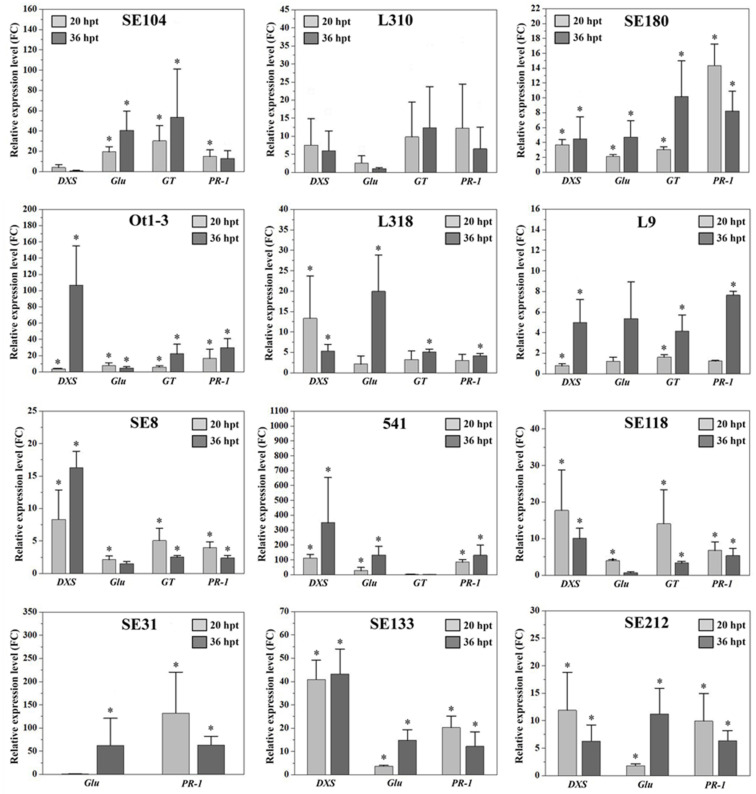
Relative *DXS*, *Glu*, *GT* and *PR-1* expression levels (fold change; FC) in 12 rye inbred lines inoculated with *Prs* or the mock control at 20 and 36 hpt. The target gene expression levels were normalised against the *Act* (actin) and *ADP* (ADP-ribosylation factor 1-like protein) expression levels at both time-points. The gene expression analysis was performed using three biological replicates, with the 2^−∆∆Ct^ method used to calculate the FC value (compared with the control). Data are presented as the mean ± standard error of the mean (SEM) from three independent experiments. The asterisk indicates a significant difference compared with the control as revealed by REST [29], (*p* < 0.05).

Following the *Prs* infection, the expression of all four analysed genes (*DXS*, *Glu*, *GT* and *PR-1*) increased compared to the mock-treated plants (Figure 3). Their expression patterns varied among the rye genotypes and time-points (20 or 36 hpt). Almost all lines responded in a genotype-specific manner. The exceptions were line SE118, in which the expression of all genes was induced at 20 hpt, and line L9, in which the expression of all genes was induced only at 36 hpt. Specific details regarding the expression of each gene are provided below and in Table S1.

### 3.3. DXS Gene

The expression of *DXS* was detected in all inbred lines, except for SE31. In lines SE104, L310, L318, SE118 and SE212, *DXS* was more highly expressed at 20 hpt than at 36 hpt. However, significant increases in expression (relative to the expression in the mock-inoculated control) were detected only in lines L318, SE118 and SE212. The expression of this gene increased significantly between 20 and 36 hpt in lines SE180, Ot1-3, L9, SE8, 541 and SE133. At both time-points, *DXS* expression peaked in inbred line 541, with 112.72 FC at 20 hpt and 350.00 FC at 36 hpt. The lowest *DXS* expression levels were observed in L9 (0.79 FC) at 20 hpt and in SE104 (0.77 FC) at 36 hpt. Relatively large increases in *DXS* expression between 20 and 36 hpt were detected in Ot1-3 (3.53 to 106 FC), L9 (0.79 to 4.98 FC), 541 (112.72 to 350.00 FC) and SE8 (8.30 to 16.29 FC). A sharp decrease in *DXS* expression between 20 and 36 hpt was observed in lines L318 (13.34 to 5.33 FC) and SE118 (17.72 to 10.05 FC). Relatively minor changes in *DXS* expression between the two time-points were detected in lines SE180 and SE133 (slight increase) as well as in line L310 (slight decrease).

### 3.4. Glu Gene

The increase in the *Glu* expression level was greater at 20 hpt than at 36 hpt in lines L310, Ot1-3, SE8 and SE118, whereas it was greater at 36 hpt than at 20 hpt in lines SE104, SE180, L318, L9, 541, SE31, SE133 and SE212. At both time-points, *Glu* expression was highest in inbred line 541, with 27.42 FC at 20 hpt and 130.89 FC at 36 hpt. In contrast, the lowest *Glu* expression levels were detected in SE31 and SE118, with 0.65 FC at 20 hpt and 0.71 FC at 36 hpt, respectively. A considerable increase in *Glu* expression between 20 and 36 hpt was detected in L318 (2.15 to 19.93 FC), L9 (1.2 to 5.37 FC), 541 (27.42 to 130.89 FC), SE104 (19.47 to 40.59 FC), SE133 (3.68 to 14.76 FC), SE31 (0.65 to 62.33 FC) and SE212 (1.79 to 11.22 FC), but a sharp decrease in *Glu* expression between the two time-points was observed in SE118 (3.99 to 0.7 FC). Relatively small changes in *Glu* expression levels between 20 and 36 hpt were observed in line SE180 (slight increase) as well as in lines Ot1-3, L310 and SE8 (slight decrease).

### 3.5. GT Gene

The *GT* gene was expressed in all inbred lines, except for SE31, SE133 and SE212. Additionally, *GT* was more highly expressed at 20 hpt than at 36 hpt in SE8, 541 and SE118, whereas it was more highly expressed at 36 hpt than at 20 hpt in SE104, L310, SE180, Ot1-3, L318 and L9. Inbred line SE104 had the highest *GT* expression level at both time-points, with 30.25 FC at 20 hpt and 53.44 FC at 36 hpt. Conversely, the lowest *GT* expression level was observed in 541 at 36 hpt (1.13 FC). The *GT* expression level increased between 20 and 36 hpt in several inbred lines, including Ot1-3 (5.81 to 22.14 FC), SE180 (3.03 to 10.17 FC) and SE104 (30.25 to 53.44 FC). In contrast, there was a marked decrease in *GT* expression between the two time-points in SE118 (14.06 to 3.33 FC). Relatively minor changes in *GT* expression between 20 and 36 hpt were detected in lines L318, L9 and L310 (slight increase) as well as in lines 541 and SE8 (slight decrease).

### 3.6. PR-1 Gene

The *PR-1* expression level was higher at 20 hpt than at 36 hpt in inbred lines SE104, L310, SE180, SE8, SE118, SE31, SE133 and SE212, while it was higher at 36 hpt than at 20 hpt in Ot1-3, L318, L9 and 541. The highest *PR-1* expression levels were observed in SE31 at 20 hpt (132.09 FC) and 541 at 36 hpt (130.42 FC). The lowest *PR-1* expression levels were detected in L9 and SE8 at 20 hpt (1.25 FC) and 36 hpt (2.39 FC), respectively. Sharp increases in *PR-1* expression were detected between 20 and 36 hpt in Ot1-3 (16.58 to 29.63 FC), L9 (1.25 to 7.64 FC) and 541 (85.43 to 130.42 FC), whereas considerable decreases in expression between the first and second time-points were observed in SE180 (14.35 to 8.23 FC), L310 (12.25 to 6.53 FC), SE133 (20.20 to 12.14 FC) and SE31 (132.09 to 63.25 FC). Small changes in the *PR-1* expression level occurred between 20 and 36 hpt in line L318 (slight increase) as well as in lines SE8, SE104, SE118 and SE212 (slight decrease).

All studied genes were upregulated during infection with *Prs*, but only in the case of the *PR-1* gene, the expression level at 36 hpt compared with the mock-treated plants was significantly correlated with infection type (Table S3).

## 4. Discussion

Plant disease resistance is associated with the activation of various defence mechanisms that delay or prevent infections at specific stages of the host–pathogen interaction. In plants, extracellular receptors act as gatekeepers, detecting incoming pathogen-associated molecular patterns (PAMPs) [30]. The recognition of PAMPs triggers a basal defence mechanism known as pattern-triggered immunity (PTI) [30,31,32], which can confer broad-spectrum resistance through a basal defence network. However, the co-evolution of pathogens and their host plants has enabled pathogens to overcome the basal defence barrier by secreting effectors that are transported into the host cells [30,33]. Plant resistance (*R*) genes provide resistance against specific strains or races of pathogens by encoding proteins that recognise specific effector molecules.

In this study, four candidate genes conferring resistance to *Prs* (*DXS*, *Glu*, *GT* and *PR-1*) were selected for an RT-qPCR analysis. According to a global transcriptome analysis [12], these genes are differentially expressed after a *Prs* infection. We analysed whether these genes provide protection against LR using a relatively large set of inbred lines (which included the three above-mentioned lines). Additionally, a microscopic examination was performed to comprehensively investigate plant–pathogen interactions.

First, we demonstrated that the expression of all four genes was upregulated in response to the *Prs* infection in almost all of the inbred lines. We were unable to detect the expression of one or two genes in only three lines. More specifically, *DXS* and *GT* were not expressed in line SE31, whereas *GT* expression was undetectable in lines SE133 and SE212. The observed lack of expression suggests these genes are relatively unaffected by the *Prs* infection or their transcription is inhibited by various unknown regulatory mechanisms. These possibilities will need to be experimentally verified. With the exception of *DXS*, the roles of the other genes in the immune response to LR have been confirmed.

The first of the analysed genes, *Glu*, encodes a β-1,3-glucanase, which is an “antifungal hydrolase” that belongs to the PR-2 family of pathogenesis-related proteins. One of its major roles in plants involves defence responses to pathogens. It degrades the cell walls of fungal pathogens by hydrolysing the β-1,3-D-glycosidic bonds, thereby weakening the pathogen structure and impeding its growth and ability to invade the plant host [34]. The role of this enzyme in wheat plant defences against LR was revealed by Gao et al. [15] and Neugebauer et al. [17]. The RT-qPCR analysis by Gao et al. [15] indicated that the expression of a Glu-encoding gene (*TcLr19Glu*) is induced by *Puccinia triticina* during compatible interactions, but more so during incompatible interactions, with peak expression levels between 24 and 48 hpt. This is consistent with the results of the current study, in which the upregulated expression of *Glu* in the plants infected with *Prs* (compared with the mock-inoculated plants) was greater at 36 hpt than at 20 hpt in most lines. However, because the opposite expression dynamics were detected in 5 of the 12 inbred lines, the relationship between the changes in *Glu* expression over time and the defence response of *Prs*-infected plants cannot be conclusively determined. Neugebauer et al. [17] infected wheat cultivar Thatcher with six *P. triticina* races and detected a gradual increase in the expression of *Glu* and genes encoding PR-1 and PR-5 thaumatin-like protein between 1 and 3 dpi, which was followed by a decrease in expression at 5 dpi and then another increase in expression at 6 dpi. The authors suggested that such changes in *Glu* expression may help protect plants from LR. To determine whether this possibility also applies to rye infected with LR, research over a longer post-infection period will need to be conducted.

The next gene, *GT*, encodes a UDP-glycosyltransferase belonging to the multigenic superfamily of enzymes found in all living organisms. GTs are associated with plant resistance to various diseases, including LR. Bolton et al. [19] identified two GT-encoding genes in wheat that are associated with *Lr34*-mediated resistance; the expression levels of both genes were revealed to be highly upregulated after a 3-day infection with *P. triticina*. A GT-encoding gene (*Ta.90050*) may be involved in the late resistance response of wheat to stem rust [20]. Amo and Soriano [18] recently identified five upregulated genes encoding GTs by conducting a meta-QTL analysis. Among these genes, *TraesCS7D02G217700* has been proposed as a candidate gene conferring resistance to LR. Furthermore, Kumar et al. [35] used high-confidence meta-QTLs to identify three GT-encoding genes (*TraesCS2B02G012000*, *TraesCS5A02G305000* and *TraesCS5A02G305100*) in wheat that may be linked to stripe rust resistance. In a previous study [10], we showed that in rye inoculated with a semi-compatible *Prs* isolate, the protective role of GT may be related to the conversion of DIBOA to DIBOA glucoside, the content of which increased at 24 hpt and then decreased at 48 hpt. It is worth noting that in our studies described here, the expression of this gene increased over time—it was higher at 36 hpt than at 20 hpt in susceptible lines (SE104, L310, SE180, Ot1-3, L318, L9) and decreased over time in two resistant lines (SE8 and SE118).

The third of the examined genes, *PR-1,* encodes the PR-1 protein, which inhibits the development of diseases (e.g., rusts) caused by many fungal pathogens [23]. In wheat, LR induces the expression of PR-1-encoding genes, namely *TcLr19PR1* [15], *TaLr35PR1* [21], *PR-1* [17] and *TaPR1* [36], which may be useful for characterising the infection type. The *PR-1* expression profiles described herein reflect the involvement of this gene in the rye immune response to LR; moreover, similarly to the *GT* gene, its expression increased over time in most susceptible lines and decreased over time in resistant lines.

The last one, the *DXS* gene, encodes the first and rate-limiting enzyme in the plastidial methylerythritol phosphate pathway, which produces isopentenyl diphosphate and its isoform dimethylallyl diphosphate as terpenoid biosynthesis precursors and plays essential roles in many physiological processes, including defence responses to pathogens [37,38]. However, there are no reports describing a relationship between *DXS* genes and plant responses to LR infections. The increased expression of this gene in 12 rye lines with different origins implies *DXS* may be important for the rye immune response to LR.

Based on macroscopic and microscopic observations, the analysed lines were divided into two groups: susceptible and resistant. Eight lines were designated as susceptible (Ot1-3, L310, 541, L9, L318, SE180, SE104 and SE31), with profile i (HMCs without micronecrosis) revealed as the most common profile, and infection types assessed as 4 and 3. Among these lines, L318 was previously identified as the most susceptible line under field conditions [11] and after an infection by *Prs* isolate 1.1.6 [10], which was also used in the current study.

Four lines (SE133, SE8, SE212 and SE118) were assessed as resistant because their infection types were 1 and 0, and the extent of micronecrosis at the infection sites was very high (profile ii and iii). Profile iii is indicative of the effective inhibition of pathogen growth. These observations are consistent with the results of a previous study by Orczyk et al. [26], in which micronecrosis was undetectable in susceptible wheat lines infected with *P. triticina* (Thatcher and *TcLr34*). In the same study, the infection sites in the most resistant lines at 3 and 4 dpi had necrotic lesions but lacked HMCs (*TcLr9*, *TcLr19* and *TcLr26*).

We observed a relatively pronounced relationship between the infection profiles and the expression of the analysed genes. In the resistant lines, the FC values were usually higher at 20 hpt than at 36 hpt, while the opposite trend was observed in the susceptible lines. This suggests the resistant lines may respond to the fungal pathogen earlier than the susceptible lines via the induced expression of a set of defence-related genes. Another interesting observation was that even though all studied genes were upregulated during infection with *Prs*, the expression level at 36 hpt was significantly correlated with infection type only in the case of the PR-1 gene. Therefore, the PR-1 gene may be useful as a resistance marker for determining the type of infection.

The analysis of the expression of four *R* gene candidates (*DXS*, *Glu*, *GT* and *PR-1*) demonstrated their importance for the rye immune response to *Prs*, which is the fungus responsible for one of the most damaging rye diseases (i.e., LR). The highly upregulated expression of these genes during the early disease development stage may reflect their critical role in PTI.

The research findings presented herein have expanded our current understanding of the genetic basis of rye resistance to LR as well as plant–pathogen interactions at the molecular level. The study data may be useful for improving agronomic practices to control fungal diseases and for developing molecular diagnostic tools facilitating germplasm selection according to the expression of LR resistance-related genes. Specifically, the *GT* and *PR-1* expression levels during the early stage of disease development (20–36 hpt) may be a good predictor of the defence potential of a given rye genotype in pre-breeding studies. Of course, it would be necessary to verify the function of these genes in field conditions, especially being aware of climate changes and the specificity of local cultivation regimes as recently highlighted by Paraschivu et al. [39].

## Figures and Tables

**Table 1:** Plant materials used in the experiments.

Inbred Line Designation	Origin
L318	Department of Plant Genetics, Breeding and Biotechnology, Warsaw University of Life Sciences, Poland
L9	
L310	
Ot1-3	Department of Plant Genetics, Breeding and Biotechnology, West Pomeranian University of Technology in Szczecin, Poland
541	
SE8	Danko Plant Breeding, Ltd. (Choryń, Poland)
SE31	
SE104	
SE118	
SE180	
SE212	
SE133	

**Table 2:** Sequences of the RT-qPCR primers.

Gene	Primer Sequences (5′ → 3′)	
	Forward Primer	Reverse Primer
*DXS*	CTTACGAGGCTCCAGTCCAG	GCGGGCATACTCATCCACTT
*Glu*	TACCAGAACCTGTTCGACGC	GCCCTTCCTGTTCTCGTTGA
*GT*	ATACGGATCCCAAGGACCGA	CTTGCATTGAATGGACCTTACCA
*PR-1*	CTGTTTCGTCGCCAAGGAGT	CCTCCAGCACCTCCATCTTG
*Act*	CCCCTTTGAACCCAAAAGCC	GAAAGCACGGCCTGAATAGC
*ADP*	TCTCATGGTTGGTCTCGATG	GGATGGTGGTGACGATCTCT

## Data Availability

All relevant data are within the paper and in the Supporting Information file. The data of RNA-seq (in fastq format) have been deposited in NCBI (http://www.ncbi.nlm.nih.gov/bioproject/888031 (accessed on 25 January 2024); BioProject accession number: PRJNA888031).

## Supplementary Materials

The following supporting information can be downloaded at https://www.mdpi.com/article/10.3390/genes15030275/s1: Table S1: Mean values of fold changes (Prs vs. mock-treated) in expression of target genes in 12 rye inbred lines; Table S2: Spearman correlations between infection type and infection profile; Table S3: Spearman correlations between infection type and Prs-induced increase in gene expression level.
